# The outcomes of patients with diabetes mellitus in The Philippine CORONA Study

**DOI:** 10.1038/s41598-021-03898-1

**Published:** 2021-12-24

**Authors:** Adrian I. Espiritu, Harold Henrison C. Chiu, Marie Charmaine C. Sy, Veeda Michelle M. Anlacan, The Philippine CORONA Study Group, Corina Maria Socorro A. Macalintal, Joanne B. Robles, Paulo L. Cataniag, Manolo Kristoffer C. Flores, Noreen Jhoanna C. Tangcuangco-Trinidad, Dan Neftalie A. Juangco, Giuliani Renz G. Paas, Audrey Marie U. Chua, Valmarie S. Estrada, Philip Rico P. Mejia, Therese Franz B. Reyes, Maria Teresa A. Cañete, Ferdinand Renfred A. Zapata, Franko Eugenio B. Castillo, Romulo U. Esagunde, Jean B. Gantioque, Maritoni C. Abbariao, Geramie M. Acebuque, Evram V. Corral, Marian Irene C. Escasura, Marissa T. Ong, Krizelle Fowler, Arnold Angelo M. Pineda, Khasmeen D. Aradani, Joseree-Ann S. Catindig, Mark Timothy T. Cinco, Mark Erving H. Ramos, Romulus Emmanuel H. Cruz, Marita B. Dantes, Norberto A. Francisco, Rosalia A. Teleg, Krisverlyn B. Bellosillo, Jean Paolo M. Delfino, Cid Czarina E. Diesta, Rosalina B. Espiritu-Picar, Julie Anne V. Gamboa, Cara Camille M. Matute, Franzelle P. Padilla, John Joshua Q. Punsalan, Ma. Epifania V. Collantes, Charmaine B. Que, Hanifa A. Sampao, Maxine Camela S. Sta. Maria, Marita M. Fuentes, Jennifer Justice F. Manzano, Rizza J. Umali, Marc Conrad C. Molina, Hazel Claire Minerva-Ang, Arturo F. Surdilla, Loreto P. Talabucon, Natasha F. Wabe, Maria Victoria G. Manuel, Al Inde John A. Pajantoy, Josephine Cecilia V. Roque, Paul Emmanuel L. Yambao, Christian Paul B. Banday, Chritopher C. Cipriano, Nehar A. Pangandaman, Avery Gail C. Wasil, Elrey P. Inocian, Jarungchai Anton S. Vatanagul, Almira Doreen Abigail O. Apor, Carissa Paz C. Dioquino, Prinz Andrew M. Dela Cruz, Maricar P. Yumul, Ma. Alma E. Carandang-Concepcion, Ma. Caridad V. Desquitado, Carl Kevin L. Julao, Dante P. Bornales, Generaldo D. Maylem, Mark Joseph F. Cuntapay, Annabelle Y. Lao-Reyes, Aileen Mae B. Lee, Nadia O. Manlegro, Dave Mar L. Pelere, Lina C. Laxamana, Diana-Lynn S. Que, Jeryl Ritzi T. Yu, Ma. Socorro C. Martinez, Alexandria E. Matic, John Angelo Luigi S. Perez, Glenn Anthony A. Constantino, Aldanica R. Olano, Liz Edenberg P. Quiles, Artemio A. Roxas, Jo Ann R. Soliven, Michael Dorothy Frances Montojo-Tamayo, Ma. Lourdes C. Joson, Jojo R. Evangelista, Ma. Clarissa B. Nuñez, Marietta C. Olaivar, Dominique Q. Perez, Mark Deneb O. Armeña, Robert A. Barja, Joshua Emmanuel E. Abejero, Maritzie R. Eribal, Ryndell G. Alava, Muktader A. Kalbi, Nasheera W. Radja, Mohammad Elshad S. Sali, Roland Dominic G. Jamora

**Affiliations:** 1 Department of Clinical Epidemiology, College of Medicine, University of the Philippines Manila, Manila, Philippines; 2 Divison of Neurology, Department of Neurosciences, College of Medicine and Philippine General Hospital, University of the Philippines Manila, Manila, Philippines; 3 Division of Endocrinology, Diabetes and Metabolism, Department of Medicine, College of Medicine and Philippine General Hospital, University of the Philippines Manila, Manila, Philippines; 4 Institute for Neurosciences, St. Luke’s Medical Center, Global City, Philippines; 5 Division of Adult Neurology, Office of the Department of Neurosciences, College of Medicine and Philippine General Hospital, University of the Philippines Manila, Taft Avenue, 1000 Ermita, Manila, Philippines; 6 Asian Hospital and Medical Center, Muntinlupa City, Philippines; 7 Baguio General Hospital and Medical Center, Baguio City, Philippines; 8 Cagayan Valley Medical Center, Tuguegarao City, Philippines; 9 Capitol Medical Center, Quezon City, Philippines; 10 Cardinal Santos Medical Center, San Juan City, Philippines; 11 Chong Hua Hospital, Cebu City, Philippines; 12 De La Salle University Medical and Health Sciences Institute, Dasmariñas City, Philippines; 13 Dr. Jose N. Rodriguez Memorial and Sanitarium Hospital, Caloocan City, Philippines; 14 Dr. Pablo O. Torre Memorial Hospital, Bacolod City, Philippines; 15 East Avenue Medical Center, Quezon City, Philippines; 16 Institute of Dementia Care Asia, Quezon City, Philippines; 17 Jose B. Lingad Memorial Regional Hospital, City of San Fernando, San Fernando, Philippines; 18 Jose R. Reyes Memorial Medical Center, Manila, Philippines; 19 Lung Center of the Philippines, Quezon City, Philippines; 20 Makati Medical Center, Makati City, Philippines; 21 Manila Doctors Hospital, Manila, Philippines; 22 Medical Center Manila, Manila, Philippines; 23 New Era General Hospital, Quezon City, Philippines; 24 Northern Mindanao Medical Center, Cagayan de Oro City, Philippines; 25 Quirino Memorial Medical Center, Quezon City, Philippines; 26 Ospital Ng Makati, Makati City, Philippines; 27 Perpetual Succour Hospital, Cebu City, Philippines; 28 Philippine General Hospital, Manila, Philippines; 29 Philippine Heart Center, Quezon City, Philippines; 30 Research Institute for Tropical Medicine, Muntinlupa City, Philippines; 31 San Juan De Dios Educational Foundation Inc.– Hospital, Pasay City, Philippines; 32 San Lazaro Hospital, Manila, Philippines; 33 Southern Isabela Medical Center, Santiago City, Philippines; 34 Southern Philippines Medical Center, Davao City, Philippines; 35 St. Luke’s Medical Center - Global City, Taguig City, Philippines; 36 St. Luke’s Medical Center, Quezon City, Philippines; 37 The Medical City, Pasig City, Philippines; 38 University of Santo Tomas Hospital, Manila, Philippines; 39 University of the East Ramon Magsaysay Memorial Medical Center Inc., Quezon City, Philippines; 40 Veterans Memorial Medical Center, Quezon City, Philippines; 41 Vicente Sotto Memorial Medical Center, Cebu City, Philippines; 42 Western Visayas Medical Center, Iloilo City, Philippines; 43 Zamboanga City Medical Center, Zamboanga City, Philippines

**Keywords:** Diabetes complications, Neurology

## Abstract

Patients diagnosed with diabetes mellitus (DM) who are infected with severe acute respiratory syndrome coronavirus 2 (SARS-COV-2) belong to the most vulnerable patient subgroups. Emerging data has shown increased risks of severe infections, increased in ICU admissions, longer durations of admission, and increased mortality among coronavirus disease 2019 (COVID-19) patients with diabetes. We performed a subgroup analysis comparing the outcomes of patients diagnosed with DM (n = 2191) versus patients without DM (n = 8690) on our data from our study based on a nationwide, comparative, retrospective, cohort study among adult, hospitalized COVID-19 patients involving 37 hospital sites from around the Philippines. We determined distribution differences between two independent samples using Mann–Whitney U and t tests. Data on the time to onset of mortality, respiratory failure, intensive care unit (ICU) admission were used to build Kaplan–Meier curves and to compute for hazard ratios (HR). The odds ratios (OR) for longer ventilator dependence, longer ICU stay, and longer hospital stays were computed via multivariate logistic regression. Adjusted hazard ratios (aHR) and ORs (aOR) with 95% CI were calculated. We included a total of 10,881 patients with confirmed COVID-19 infection (2191 have DM while 8690 did not have DM). The median age of the DM cohort was 61, with a female to male ratio of 1:1.25 and more than 50% of the DM population were above 60 years old. The aOR for mortality was significantly higher among those in the DM group by 1.46 (95% CI 1.28–1.68; *p* < 0.001) as compared to the non-DM group. Similarly, the aOR for respiratory failure was also significantly higher among those in the DM group by 1.67 (95% CI 1.46–1.90). The aOR for developing severe COVID-19 at nadir was significantly higher among those in the DM group by 1.85 (95% CI 1.65–2.07; *p* < 0.001). The aOR for ICU admission was significantly higher among those in the DM group by 1.80 (95% CI 1.59–2.05) than those in the non-DM group. DM patients had significantly longer duration of ventilator dependence (aOR 1.33, 95% CI 1.08–1.64; *p* = 0.008) and longer hospital admission (aOR 1.13, 95% CI 1.01–1.26; *p* = 0.027). The presence of DM among COVID-19 patients significantly increased the risk of mortality, respiratory failure, duration of ventilator dependence, severe/critical COVID-19, ICU admission, and length of hospital stay.

## Introduction

The coronavirus disease 2019 (COVID-19) is caused by severe acute respiratory syndrome coronavirus 2 (SARS-CoV-2) and has affected over 150 million individuals worldwide as of July 19, 2021^1^. During this period in the Philippines, our data showed a total of 1.5 million confirmed total cases; nearly 50 thousand are active cases and over 27 thousand deaths from this infection^2^.

Patients diagnosed with diabetes mellitus (DM) in COVID-19 are among the most vulnerable. In a meta-analysis, DM was shown to be second to hypertension as the most prevalent comorbidity among patients hospitalized with COVID-19, with prevalence ranging from 8.0 to 9.7%^3^. Studies have shown that regardless of the type of DM, these patients have 50% higher risks of acquiring lower respiratory tract infections^4,5^. Among patients with DM infected with COVID-19, several mechanisms have been postulated that can contribute to increased susceptibility: (a) impaired neutrophil recruitment, (b) impaired macrophage activity, (c) impaired interferon-gamma production and release from natural killer cells, (d) impairment of antigen presentation resulting in a dysregulated immune response, cytokine storm and systemic inflammation^6^, and (e) increased angiotensin converting enzyme-2 (ACE-2) expression, a surface receptor expressed by epithelial cells of the lung, intestine, kidney and blood vessels causing vasodilation, and hypotension^7^.

Infection with SARS-CoV2 virus triggers a higher degree of stress resulting in greater release of counterregulatory hormones and higher levels of insulin resistance, all of which leads to hyperglycemia^8^. Various studies have demonstrated overall poorer outcomes among patients with DM infected with COVID-19^9–16^, these include increased mortality, respiratory failure, severity of COVID-19, increased utilization of intensive care, longer intensive care unit (ICU) stay, and prolonged hospital stay^16–18^. Thus, early identification of patients with DM will lead to more effective management of hyperglycemia towards improving outcomes^16–22^.

As COVID-19 is still presently a global pandemic, more research is needed to provide a better understanding of the effects of underlying comorbidities especially DM on COVID-19 in the setting of a low to middle income country such as the Philippines where access to healthcare services remains to be one of the major barriers in DM care. Hence, our main objective was to determine and compare the outcomes of COVID-19 patients with DM versus those without DM history in terms of COVID-19 severity, mortality, respiratory failure, COVID-19 severity, ICU admission, length of ICU stay, and length of hospital stay as these will greatly impact clinical management and patient outcomes.

## Methods

### Study design

We performed an analysis of patients diagnosed with DM based on the data from a nationwide, multicenter, comparative, retrospective, cohort study involving patients with COVID-19 who were admitted to our hospitals/study sites from February 2020 until December 2020^23,24^. All methods were carried out in accordance with relevant guidelines. The study’s protocol was registered in ClinicalTrials.gov (NCT04386083). The study was approved and endorsed by the Single Joint Research Ethics Board of the Department of Health, Philippines (SJREB-2020–24) and the independent hospital institutional review boards (see Appendix 1). All the institutional review boards mentioned waived the need for informed consent since this study used a method of naturalistic observation. The data was obtained only though review of medical records retrospectively. Moreover, the data collection forms used did not contain any information that could determine or identify the patients.

### Setting

The Philippine CORONA (COVID-19 Outcomes: a Retrospective study Of Neurological manifestations and Associated symptoms) study encompassed a total of 37 major hospitals/study sites from various regions in the Philippines (see Appendix 2)^23,24^.

### Data collection, patient selection, sampling and cohort description

We conducted a nationwide, multicenter study involving 37 institutions in the Philippines. The cases were identified using the designated COVID-19 censuses of all the participating centers. A total enumeration of patients admitted with confirmed COVID-19 disease with a discharge disposition was done in this study. The following pertinent data were obtained through review of medical records and encoded using an electronic data collection form using Epi Info Software (V.7.2.2.16): (a) demographic data; (b) other clinical profile data/comorbidities; (c) neurological history; (d) date of illness onset; (e) respiratory and constitutional symptoms associated with COVID-19; (f) COVID-19 disease severity at nadir; (g) data if neurological manifestation/s were present at onset prior to respiratory symptoms and the specific neurological manifestation/s present at onset; (h) neurological symptoms; (i) date of neurological symptom onset; (j) new-onset neurological disorders or complications; (k) date of new neurological disorder or complication onset; (l) imaging done; (m) cerebrospinal fluid analysis; (n) electrophysiological studies; (o) treatment given; (p) antibiotics given; (q) neurological interventions given; (r) date of mortality and cause/s of mortality; (s) date of respiratory failure onset, date of mechanical ventilator cessation and cause/s of respiratory failure; (t) date of first day of ICU admission, date of discharge from ICU and indication/s for ICU admission; (u) other neurological outcomes at discharge; (v) date of hospital discharge; and (w) final disposition.

We included all patients analyzed in The Philippine CORONA Study^23,24^. Patients with DM were identified through history of having diabetes mellitus diagnosed using any of the following criteria: fasting blood glucose of ≥ 126 mg/dL, hemoglobin A1c (HbA1c) of ≥ 6.5% and random blood sugar of ≥ 200 mg/dL in the presence of symptoms of hyperglycemia. Adult COVID-19 patients who had DM were grouped under the exposed cohort while those without DM (non-DM) were classified under the unexposed cohort.

### Outcome variables

We obtained the following relevant patient outcomes: (a) mortality; (b) COVID-19 severity at nadir defined as the worst covid classification of severity the patient experienced during the admission, disease severity was defined as mild: defined as presence of mild pneumonia or absence of pneumonia; severe disease: defined as the presence of dyspnea, respiratory rate > 30 breaths/minute, hypoxia (SpO2 < 93%) or > 50% lung involvement on imaging within 24–48 h; and critical disease: defined as the presence of respiratory failure, shock or multiorgan dysfunction; (c) respiratory failure (patients with clinical symptoms/signs of respiratory insufficiency defined as increased work of breathing/tachypnea [respiratory rate > 22], a necessity to administer supplemental oxygen, or abnormal blood gases [partial pressure of oxygen < 60/ hypoxemia or partial pressure of carbon dioxide > 45/ hypercapnia]); (d) duration of ventilator dependence (DVD) (days from the start of assisted ventilation to cessation); (e) ICU admission (COVID-19 patients admitted to an ICU or ICU-comparable setting; (f) length of ICU stay (LICUS) (days admitted in the ICU); and (g) length of hospital stay (LHS) (days from admission to discharge).

### Sample size

The total sample of patients analyzed was 10,881: 2191 have DM versus 8690 without DM^24^.

### Statistical analysis

Baseline characteristics and clinical outcomes of the participants were summarized by descriptive statistics. Numerical variables were described as mean and standard deviation (SD), if the data was normally distributed as assessed by Shapiro–Wilk test for normality, and as median and interquartile range (IQR), if otherwise. Categorical variables were described as counts and proportions. These different baseline characteristics and clinical outcomes were compared between diabetics and non-diabetics. Significant difference in the mean/median/mean-rank of the different numerical variables was determined by Student’s t test for the variables with normally distributed data, while Mann–Whitney U test was done for non-normally distributed variables. Heterogeneity of the proportions of the different categorical variables was determined by chi-square test or Fisher exact test.

The associations between history of DM and the different individual dichotomous outcome variables of interest were determined by multivariable binary logistic regression. Survival analysis was also done for time-to-event data of mortality, respiratory failure, and admission to ICU. The time-to-event were right-censored on time-to-discharge as the exit from the time-at-risk among those who have not experienced the event, i.e., mortality or respiratory failure, or admission to ICU, during the hospital stay. The associations between history of DM and the different time-to-event outcome variables of interest were determined by multivariable Cox proportional hazards regression. The logistic regression and Cox proportional hazards models were adjusted for the following pre-determined confounders: age group, sex, smoking status, hypertension, chronic cardiac disease, chronic respiratory disease, chronic kidney disease, chronic neurologic disease, chronic liver disease, and HIV/AIDS. A cutoff of *p*-value < 0.05 identifies history of DM as significant predictor of the different outcomes of interest.

Kaplan–Meier curves were constructed to visualize the survival curves of DM versus non-DM, and log-rank test was used to identify significant differences in the survival curves across between diabetics and non-diabetics; log-rank test with *p*-value < 0.05 was considered significant. All statistical analyses were conducted using Stata®, Version 7.2.2.16 (College Station, TX: StataCorp LP).

## Results

### Inclusion of patients

We identified hospitalized patients diagnosed with DM and COVID-19 verified via reverse transcription polymerase chain reaction from the 37 participating study sites. A total of 10,881 were included in the qualitative and quantitative analyses. Two thousand one hundred and ninety-one patients were identified with the primary exposure (DM group) while the remaining 8690 did not have diabetes (non-DM).

### Demographic and clinical characteristics of included COVID-19 patients with diabetes mellitus

The median age of the DM cohort was 61 with a female to male ratio of 1:1.25. More than 50% of the diabetic population were more than 60 years old (n = 1218, 55.6%). Hypertension (HPN) (n = 1643, 75.0%), HIV/AIDS (n = 460, 20.1%), and chronic respiratory disease (n = 297, 13.5%) were the most common comorbidities. A considerable proportion of patients received systemic glucocorticoids (n = 1105, 50.4%), remdesivir (n = 769, 35.1%), and tocilizumab (n = 464, 21.18%). Other pertinent clinical features and comparison of characteristics are presented in Table 1.

**Table 1:** Baseline characteristics stratified by history of diabetes.

	Diabetic	Non-diabetic	*p*-value
	(n = 2191)	(n = 8690)	
**Socio-demographic data**			
**Age, median (IQR)**	61 (18)	48 (29)	< 0.001
**Age group**			
18 – 59 years, n (%)	973 (44.41%)	6074 (69.90%)	< 0.001
≥ 60 years, n (%)	1218 (55.59%)	2616 (30.10%)	
**Female, n (%)**	975 (44.50%)	4124 (47.47%)	0.013
**Ever-smoker (past/current), n (%)**	321 (14.65%)	705 (8.11%)	< 0.001
**Clinical characteristics**			
**Non-neurologic comorbidities**			
Hypertension, n (%)	1643 (74.99%)	2004 (23.06%)	< 0.001
Chronic cardiac disease^a^, n (%)	185 (8.44%)	431 (4.96%)	< 0.001
Chronic respiratory disease^b^, n (%)	297 (13.56%)	314 (3.61%)	< 0.001
Chronic kidney disease, n (%)	22 (1.00%)	38 (0.44%)	0.001
Chronic liver disease, n (%)	64 (2.92%)	180 (2.07%)	0.016
Malignancy, n (%)	3 (0.14%)	34 (0.39%)	0.068
HIV/AIDS, n (%)	460 (20.99%)	1279 (14.72%)	< 0.001
**Number of non-neurologic comorbidities, median (IQR)**	2 (1)	0 (1)	< 0.001
**Non-neurologic presenting symptom, n (%)**	2029 (92.61%)	5037 (57.96%)	< 0.001
Fever, n (%)	1301 (59.38%)	2626 (30.22%)	< 0.001
Cough, n (%)	1444 (65.91%)	2967 (34.14%)	< 0.001
Dyspnea, n (%)	948 (43.27%)	1755 (20.20%)	< 0.001
Rhinorrhea, n (%)	123 (5.61%)	484 (5.57%)	0.936
Sputum production, n (%)	214 (9.77%)	423 (4.87%)	< 0.001
Sore throat, n (%)	155 (7.07%)	596 (6.86%)	0.722
Diarrhea, n (%)	150 (6.85%)	447 (5.14%)	0.002
Fatigue, n (%)	239 (10.91%)	474 (5.45%)	< 0.001
Others, n (%)	375 (17.12%)	1299 (14.95%)	0.012
**Treatment/s received**			
Glucocorticoids, n (%)	1105 (50.43%)	1739 (20.01%)	< 0.001
Tocilizumab, n (%)	464 (21.18%)	565 (6.50%)	< 0.001
Antiviral^c^, n (%)	769 (35.10%)	1133 (13.04%)	< 0.001
Antibacterial, n (%)	2042 (93.20%)	6972 (80.23%)	< 0.001
Others^d^, n (%)	987 (45.05%)	2918 (33.58%)	< 0.001

^a^Includes heart failure, coronary artery disease, prior history of myocardial infarction, and other cardiac conditions.

^b^Includes bronchial asthma, chronic obstructive pulmonary disease (COPD), restrictive lung disease, and other pulmonary conditions.

^c^Includes remdesivir, lopinavir, ritonavir.

^d^Includes chloroquine, hydroxychloroquine, convalescent plasma, and other therapies.

### Effects of diabetes mellitus on outcomes of included COVID-19 patients and survival analysis

**Figure 1:**
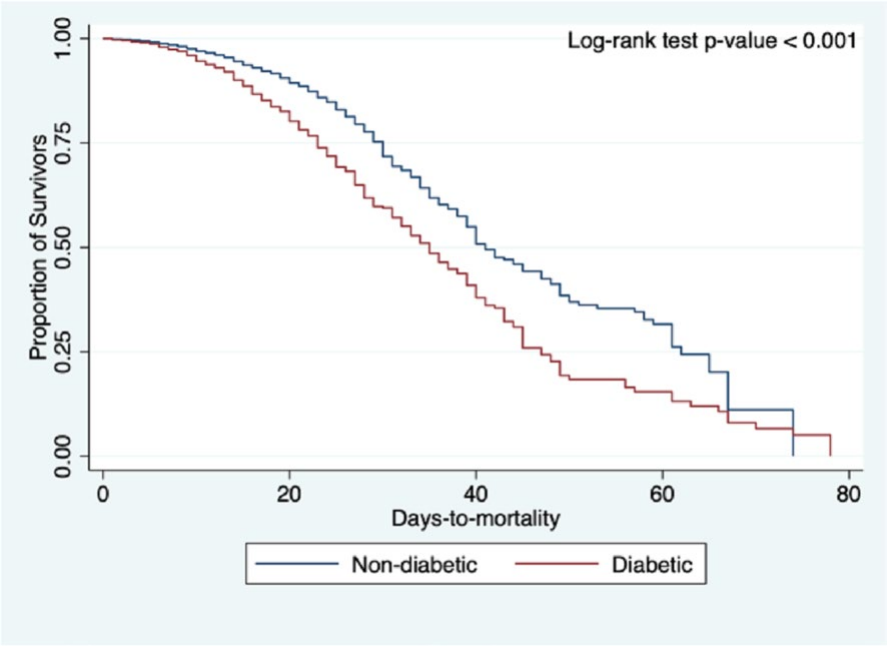
Comparison of Kaplan–Meier curves of in-hospital mortality between COVID-19 patients with diabetes versus non-diabetics, adjusted for the different confounding variables of interest.

**Figure 2:**
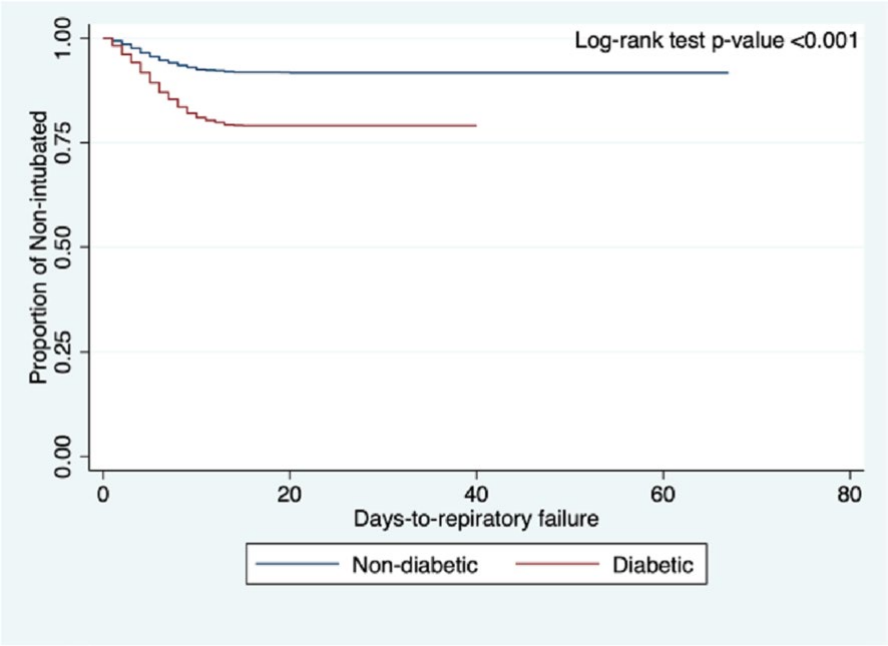
Comparison of Kaplan–Meier curves of respiratory failure between COVID-19 patients with diabetes versus non-diabetics, adjusted for the different confounding variables of interest.

**Figure 3:**
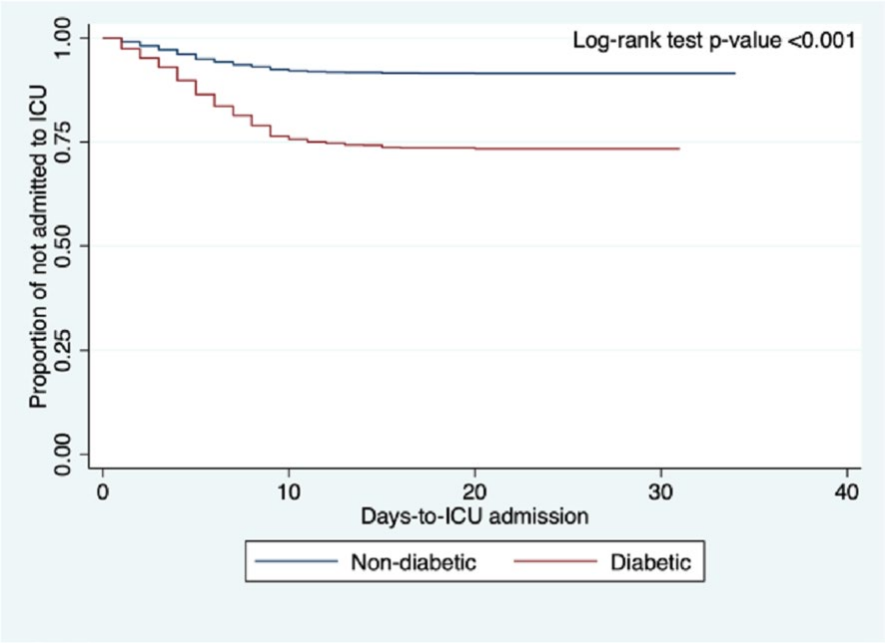
Comparison of Kaplan–Meier curves of being admitted to ICU between COVID-19 patients with diabetes versus non-diabetics, adjusted for the different confounding variables of interest.

The comparison of clinical outcomes between COVID-19 patients with DM versus those without DM are shown in Table 2. The adjusted odds ratio for mortality, respiratory failure, COVID-19 severity, ICU admission, length of ICU admission, and length of hospital stay are shown in Table 3. The adjusted odds ratio for the time-to-event analysis for mortality, respiratory failure, and ICU admission are shown in Table 4. Figures 1, 2, and 3 shows the Kaplan–Meier cumulative hazard functions for DM and non-DM groups in terms of mortality, respiratory failure, and ICU admission, respectively.

**Table 2:** Comparison of clinical outcomes in COVID-19 patients with diabetes mellitus vs. without diabetes mellitus.

Outcomes	Diabetic	Non-diabetic	*p*-value
	(n = 2191)	(n = 8690)	
**Final outcome**			
In-hospital mortality, n (%)	579 (26.43%)	1123 (12.92%)	< 0.001
Discharged, n (%)	1612 (73.57%)	7567 (87.08%)	< 0.001
**Time to in-hospital mortality in days, median (IQR)**	16 (14)	14 (13)	< 0.001
**Respiratory failure, n (%)**	656 (29.94%)	952 (10.96%)	< 0.001
Duration of IMV in days, median (IQR)	14 (12)	12 (11)	0.002
IMV dependence < 14 days, n (%)	314 (47.87%)	530 (55.79%)	0.002
IMV dependence ≥ 14 days, n (%)	342 (52.13%)	420 (44.21%)	
**COVID-19 severity at nadir**			
Mild/moderate, n (%)	923 (42.57%)	5767 (67.19%)	< 0.001
Severe/critical, n (%)	1245 (57.43%)	2816 (32.81%)	
**Admitted to ICU, n (%)**	739 (33.73%)	1001 (11.52%)	< 0.001
Length of ICU stay in days, median (IQR)	15 (11)	14 (12)	0.596
ICU stay ≤ 7 days, n (%)	119 (16.10%)	153 (15.28%)	0.642
ICU stay > 7 days, n (%)	620 (83.90%)	848 (84.72%)	
**Length of hospital stay**^a^ **in days, median (IQR)**	14 (10)	13 (9)	< 0.001
Hospital stay ≤ 14 days, n (%)	1209 (55.18%)	5368 (61.77%)	< 0.001
Hospital stay > 14 days, n (%)	982 (44.82%)	3322 (38.23%)	
**Neurologic outcome on discharge**^b^			
Full/partial neurologic recovery, n (%)	368 (76.67%)	1271 (89.19%)	< 0.001
No recovery, n (%)	112 (23.33%)	154 (10.81%)	

^a^Derived from overall length of stay for patients who were never admitted to ICU; excludes ICU length of stay for those who were admitted in the ICU.

^b^Patients who had a neurologic presentation or concomitant acute neurologic diagnosis on admission (n = 2291).

**Table 3:** Association of history of diabetes to the different outcomes of interest.

Outcomes*	Adj. OR*	95% CI	*p*-value
Severe/critical COVID-19 at nadir	1.85	1.65, 2.07	< 0.001
**Neurological presentation/complication**	1.17	1.03, 1.31	0.013
Full/partial neurological improvement	1.45	0.88, 2.38	0.145
In-hospital mortality	1.46	1.28, 1.68	< 0.001
**Respiratory failure**	1.67	1.46, 1.9	< 0.001
IMV dependence ≥ 14 days	1.33	1.08, 1.64	0.008
**ICU admission**	1.80	1.59, 2.05	< 0.001
ICU stay > 7 days	0.92	0.7, 1.21	0.553
Hospital stay > 14 days	1.13	1.01, 1.26	0.027

*Individual univariate multiple logistic regression analysis with independent variable diabetes adjusted for age group, sex, smoking history, hypertension, chronic cardiac disease, chronic kidney disease, chronic respiratory disease, chronic neurologic disease, chronic liver disease, and HIV/AIDS.

**Table 4:** Association of history of diabetes to the different outcomes of interest (time-to-event analysis).

Outcomes*	Adj. HR*	95% CI	*p*-value
In-hospital mortality	1.19	1.06, 1.33	0.002
Respiratory failure	1.51	1.35, 1.69	< 0.001
ICU admission	1.57	1.41, 1.74	< 0.001

*Individual univariate multiple Cox proportional hazards regression analysis with independent variable diabetes adjusted for age group, sex, smoking history, hypertension, chronic cardiac disease, chronic kidney disease, chronic respiratory disease, chronic neurologic disease, chronic liver disease, and HIV/AIDS.

### Mortality

A total of 1702 (15.6%) died in the full cohort. Patients with DM had a significantly higher in-hospital mortality rate (n = 579, 26.4%) compared to those without DM (n = 1123, 12.9%; *p* < 0.001). The adjusted OR for mortality was significantly higher among those in the DM group by 1.46 (95% CI 1.28–1.68; *p* < 0.001) than those in the non-DM group (see Table 3). Furthermore, time-to-event analysis showed an adjusted HR remaining significantly higher by 1.19 (95% CI 1.06–1.33; *p* = 0.002) in the DM group compared to those in the non-DM group (see Table 4, Fig. 1).

### Respiratory failure

A total of 1608 (14.8%) patients had respiratory failure. A higher proportion of patients with DM had respiratory failure (n = 656, 29.9%) as compared to the non-DM group (n = 952, 10.9%, *p* < 0.001). The adjusted OR for respiratory failure was significantly higher among the DM group by 1.67 (95% CI 1.46–1.90) than in the non-DM group (Table 3). Furthermore, time-to-event analysis showed an adjusted HR remaining significantly increased by 1.51 (95% CI 1.35–1.69; *p* < 0.001) in the DM group than those in the non-DM group.

### Duration of ventilator dependence (DVD)

Among patients needing mechanical ventilation, the overall median DVD was 13 days (IQR 8–20). There was a significant difference in the median DVD between patients with DM (median of 14 days) and non-DM groups (median of 12 days; *p* = 0.002). The adjusted OR for longer DVD (i.e., more than 14 days) was statistically significant at 1.33 (95% CI 1.08–1.64; *p* = 0.008).

### Severe/critical COVID-19 at Nadir

A significantly higher proportion of patients with DM had severe/critical COVID-19 (n = 1245, 57.4%) as compared to those without DM (n = 2816, 32.8%; *p* < 0.001). The adjusted OR for severe COVID-19 was significantly higher in the DM group by 1.85 (95% CI 1.65–2.07; *p* < 0.001) than those in the non-DM group (Table 3).

### ICU admission

A total of 1740 patients (16.0%) from our full cohort were admitted to the ICU. A significantly higher proportion of patients with DM were admitted into the ICU (n = 739, 33.7%) as compared to those without (n = 1001, 11.5%; *p* < 0.001). The adjusted OR for ICU admission was significantly higher in the DM group by 1.80 (95% CI 1.59–2.05) than in the non-DM group (see Table 3). Furthermore, the time-to-event analysis showed an adjusted HR remaining significantly increased by 1.57 (95% CI 1.41–1.74; *p* < 0.001) in the DM group as compared to the non-DM group.

### Length of ICU stay (LICUS)

Among those patients admitted to the ICU, the overall median LICUS was 15 days (IQR 9.5– 21). There was no significant difference in median LICUS between patients in the DM (median of 15 days) and non-DM groups (median of 14 days; *p* = 0.596). The adjusted OR for longer LICUS (i.e., more than 7 days) was not significant at 0.92 (95% CI 0.70–1.21; *p* = 0.553).

### Length of hospital stay (LHS)

Among the full cohort, the overall median LHS was 13 days (IQR 10–19). There was a significant difference in the median LHS between patients in the DM (median of 14 days) and non-DM groups (median of 13 days; *p* < 0.001). The adjusted OR for longer LHS (i.e., more than 14 days) was statistically significant at 1.13 (95% CI 1.01–1.26; *p* = 0.027).

## Discussion

This is the largest cohort study in the Philippines involving 10,881 hospitalized patients with COVID-19 infection and 2191 of this cohort had a history of DM with substantial information on their clinical characteristics. Furthermore, we investigated the effects of the presence of DM on clinically relevant outcomes in these patients.

Patients with DM comprised 20.1% of the entire cohort. This was consistent with studies done in China where 16.2% of patients admitted for COVID-19 also had DM^22^. Within the DM cohort, a considerable number experienced respiratory failure (29.94%), were admitted to the ICU (33.7%), or died (26.4%). The presence of DM as a comorbidity in COVID-19 patients significantly increased the risk of poor outcomes such as mortality, respiratory failure, longer duration of ventilator dependence, ICU admission, COVID-19 severity, and increased length of hospital stay compared to those patients without the disease. On the other hand, the presence of DM was not significantly associated with the length of ICU stay.

Our data showed that with adjustments for age, sex, comorbidities and taking into account the disease severity, the presence of DM increased the odds of dying by 46%. Our gross mortality rates were 26% and 13% for DM and non-DM patients, respectively, both of which were comparable to the data from a multicenter, retrospective study conducted in the United States that looked into the glycemic characteristics and clinical outcomes, where the mortality rate was 28.8% in 184 DM and/or uncontrolled hyperglycemia patients, compared with only 6.2% of 386 patients without DM^16^. The possible reason for the higher mortality in our cohort could be due to poor glycemic control, poor adherence to medications, and less access to diabetes care. A retrospective analysis of 29 patients diagnosed with type 2 DM and laboratory confirmed COVID-19 revealed that two thirds of admitted patients have uncontrolled blood glucose levels^14^, a small number of these presented with either diabetic ketoacidosis or hyperglycemic hyperosmolar state^15^. In a multicenter study in the United States, persistent hyperglycemia of more than 180 mg/dL was associated with a four-fold increase in risk for mortality in patients with COVID-19, rising up to seven-fold among those with pre-existing DM^16^. This was further supported by a meta-analysis done in Italy that compared ICU admissions and mortality among diabetic COVID-19 patients which demonstrated that diabetic patients were at a higher mortality risk (OR 3.21, 95% CI 1.82–5.64, *p* < 0.0001)^20^. The presence of DM has always been associated with a poorer prognosis in any infectious disease, but data on its effect in COVID-19 patients remains to be limited. Current data seems to indicate that diabetic patients, as would be expected, have a higher risk of developing more severe complications and higher frequency of ICU admissions^20^. Multiple mechanisms have been postulated to explain the relationship between hyperglycemia and immune dysfunction. These include impairment of white blood cell chemotaxis, phagocytosis, complement function and cytokine dysregulation^6,7^. Furthermore, immunohistochemical studies of cadaveric pancreatic islet cells revealed similar ACE 2 immunostaining pattern to those found in lung alveolar epithelium and myocardium suggesting that ACE2 expression in the pancreas caused by SARS-COV2 invasion and injury leads to pancreatitis resulting in hyperglycemia and mortality^9,25^﻿. Emerging data on the association between COVID-19 infection and DM have demonstrated that patients diagnosed to have DM have the same probability as the general population in terms of getting infected with COVID-19. However, patients with DM have higher risks of serious complications brought about by COVID-19, and the risks are similar for both type 1 and type 2 DM mellitus^12^. Historical data from the SARS-CoV pandemic from 2002 to 2003, influenza A (H1N1) pandemic in 2009 and Middle East respiratory syndrome coronavirus pandemic in 2012 have all shown poor outcomes among patients with hyperglycemia and poor glycemic control^13^.

In our cohort, we also provided evidence that the presence of DM in COVID-19 infection significantly increased the odds of developing respiratory failure by as much as 67%. DM patients also have 33% increased odds of being dependent on ventilator for more than 14 days than non-DM patients. Our study was also able to shed light on relevant evidence relating DM and severity of infection. Patients with DM have 85% increased odds of having severe/critical COVID-19 at nadir than non-diabetics. Further association studies have shown that there was a 2.26 times increase in the risk of severe infections among COVID-19 with DM resulting in worse overall outcomes^17^. This is supported by a meta-analysis of 1936 COVID-19 patients across the world, which showed a significant correlation between COVID-19 severity and the presence of DM (OR 2.67, 95% CI 1.91–3.74, *p* < 0.01)^18^. The number of comorbidities was also identified as a significant risk factor for intensive care unit (ICU) admission and a significant predictor of mortality^19–22^. Retrospective studies have illustrated the hazard ratios of ICU admission, requirement for invasive mechanical ventilation, and death among those with DM (HR 1.59, 95% CI 1.03–2.45). The HR was 1.79 (95% CI 1.16–2.77) among patients with at least one comorbidity and 2.59 (95% CI 1.61–4.17) among patients with two or more comorbidities^26^. Mortality data from the Chinese Center for Disease Control showed an overall case-fatality rate of 2.3% (1023 deaths in 44,672 cases) in the general population with an increase to 7.3% among those diagnosed with DM^27^. Diabetes, hyperlipidemia, nonalcoholic fatty liver, and atherosclerotic cardiovascular disease often occur together in a single patient and these comorbidities are deeply interrelated. Hence, the new concept of Glucolipid Metabolic Disorders (GLMD) was proposed. Among severe COVID-19 patients, over activation of T cells leads to severe immune-mediated injury resulting in higher concentrations of pro-inflammatory cytokines ending in a cytokine storm that is associated with disease severity. It has been hypothesized that this chronic inflammatory milieu in GLMD patients becomes more activated following COVID-19 infection^28^.

There was a significantly increased odds (i.e., 80% increased odds of being admitted to ICU than non-DM) in COVID-19 patients with DM compared to those without DM. This was consistent with the study done in Italy where DM patients were at a significantly increased risk of ICU admission (OR: 2.79, 95% CI 1.85–4.22, *p* < 0.0001)^20^.

Lastly, our results have shown that patients with DM have 13% increased odds of being admitted for > 14 days (median of 14 days). Among 493 discharged survivors in the United States, the median length of stay was longer in 184 patients with DM compared with 386 patients without DM or hyperglycemia (5.7 vs 4.3 days, *p* < 0.001)^16^.

Our study has several limitations. Our data only reflected exposures and outcomes of hospitalized COVID-19 patients thus, the information from patients who were not admitted was not captured. Moreover, because only admitted patients were involved in this study, mortality and respiratory failure are expected to be overestimated since substantially more severe and critical COVID-19 cases are admitted in a hospital setting. We were also not able to account for the higher utilization of steroids, tocilizumab, and anti-viral medications in our DM cohort. The duration, type of DM, presence of microvascular and macrovascular complications, and history of diabetic emergencies were not determined, nor was the level of glycemic control (fasting blood glucose levels and baseline hemoglobin A1c levels), and the treatment regimens of the patients. Future studies focusing on the aforementioned parameters are needed to further establish the role and interaction between the presence of DM, DM complications and the level of glycemic control on its effect on clinical outcomes among COVID-19 patients.

## Conclusion

The presence of diabetes mellitus among COVID-19 patients significantly increases the risk of mortality, respiratory failure, duration of ventilator dependence, severe/critical COVID-19, ICU admission, and length of hospital stay.

## Supplementary Information

Supplementary Information.
