# Supplementary material for: The outcomes of patients with diabetes mellitus in The Philippine CORONA Study
Source: Sci Rep. 2021 Dec 24;11:24436. doi: 10.1038/s41598-021-03898-1 (PMC8709842; doi:10.1038/s41598-021-03898-1)
Supplement: Supplementary file 1 — Supplementary Information. [file 41598_2021_3898_MOESM1_ESM.docx]

**Appendix 1: Ethical approval.**

The Philippine CORONA Study protocol was endorsed by the Single Joint Research Ethics Board of the Department of Health, Philippines (SJREB-2020–24) for which the following institutions were included: Cagayan Valley Medical Center, Tuguegarao City; Jose R. Reyes Memorial Medical Center, Manila; Ospital ng Makati, Makati City; Perpetual Succour Hospital, Cebu City; Philippine Heart Center, Quezon City; Southern Isabela Medical Center, Santiago City; Southern Philippines Medical Center, Davao City; Western Visayas Medical Center, Iloilo City; and Zamboanga City Medical Center, Zamboanga City. Moreover, the authors were able to receive approval from the following local institutional review boards (code): Asian Hospital and Medical Center, Muntinlupa City (2020- 010-A); Baguio General Hospital and Medical Center, Baguio City (BGHMC-ERC-2020–13); Capitol Medical Center, Quezon City; Cardinal Santos Medical Center, San Juan City (CSMC REC 2020–020); Chong Hua Hospital, Cebu City (IRB 2420–04); De La Salle Medical and Health Sciences Institute, Cavite (2020–23-02-A); Dr. Jose N. Rodriguez Memorial and Sanitarium Hospital, Caloocan City; Dr. Pablo O. Torre Memorial Hospital (Riverside Medical Center, Inc.), Bacolod City; East Avenue Medical Center, Quezon City (EAMC IERB 2020- 38); Jose B. Lingad Memorial Regional Hospital, City of San Fernando, Pampanga; Lung Center of the Philippines, Quezon City (LCP-CT-010–2020); Manila Doctors Hospital, Manila (MDH IRB 2020–006); Makati Medical Center, Makati City (MMC IRB 2020–054); Medical Center Manila, Manila (MMERC 2020–09); New Era General Hospital, Quezon City; Northern Mindanao Medical Center, Cagayan de Oro City (025–2020); Quirino Memorial Medical Center, Quezon City (QMMC REB GCS 2020–28); Philippine General Hospital, Manila (2020–314-01 SJREB); Research Institute for Tropical Medicine, Muntinlupa City (RITM IRB 2020–16); San Lazaro Hospital, Manila; San Juan De Dios Educational Foundation Inc. Hospital, Pasay City (SJRIB 2020–0006); Southern Isabela Medical Center, Santiago City (2020–03); Southern Philippines Medical Center (SPMC), Davao City (P20062001); St. Luke’s Medical Center, Quezon City (SL–20116); St. Luke’s Medical Center, Bonifacio Global City, Taguig City (SL–20116); The Medical City, Pasig City; University of the East Ramon Magsaysay Memorial Medical Center, Inc, Quezon City (0835/E/2020/063); University of Santo Tomas Hospital, Manila (UST-REC-2020–04-071-MD); Veterans Memorial Medical Center, Quezon City (VMMC- 2020–025); Vicente Sotto Memorial Medical Center, Cebu City (VSMMC-REC-O-2020–048).

**Appendix 2: Study sites.**

Asian Hospital and Medical Center, Muntinlupa City; Baguio General Hospital and Medical Center, Baguio City; Cagayan Valley Medical Center, Tuguegarao City; Capitol Medical Center, Quezon City; Cardinal Santos Medical Center, San Juan City; Chong Hua Hospital, Cebu City; De La Salle University Medical and Health Sciences Institute, Dasmariñas City; East Avenue Medical Center, Quezon City; Jose B. Lingad Memorial Regional Hospital, San Fernando, Pampanga; Dr. Jose N. Rodriguez Memorial and Sanitarium Hospital, Caloocan City; Jose R. Reyes Memorial Medical Center, Manila; Lung Center of the Philippines, Quezon City; Manila Doctors Hospital, Manila; Medical Center Manila, Manila; Makati Medical Center, Makati City; New Era General Hospital, Quezon City; Northern Mindanao Medical Center, Cagayan de Oro City; Quirino Memorial Medical Center, Quezon City; Ospital ng Makati, Makati City; Perpetual Succour Hospital, Cebu City; Dr. Pablo O. Torre Memorial Hospital (Riverside Medical Center, Inc.), Bacolod City; University of the Philippines – Philippine General Hospital, Manila; Philippine Heart Center, Quezon City; Research Institute for Tropical Medicine, Muntinlupa City; San Lazaro Hospital, Manila; San Juan De Dios Educational Foundation Inc. Hospital, Pasay City; Southern Isabela Medical Center, Santiago City; St. Luke’s Medical Center, Quezon City; St. Luke’s Medical Center, Bonifacio Global City, Taguig City; Southern Philippines Medical Center, Davao City; The Medical City, Pasig City; University of the East Ramon Magsaysay Memorial Medical Center, Inc., Quezon City; University of Santo Tomas Hospital, Manila; Veterans Memorial Medical Center, Quezon City; Vicente Sotto Memorial Medical Center, Cebu City; Western Visayas Medical Center, Iloilo City; Zamboanga City Medical Center, Zamboanga City.
